# Phospholipase Cη2 Activation Redirects Vesicle Trafficking by Regulating F-actin*

**DOI:** 10.1074/jbc.M115.658328

**Published:** 2015-10-02

**Authors:** Masaki Yamaga, D. Michelle Kielar-Grevstad, Thomas F. J. Martin

**Affiliations:** From the Department of Biochemistry, University of Wisconsin, Madison, Wisconsin 53706

**Keywords:** actin, exocytosis, membrane trafficking, phosphoinositide, phospholipase C, vesicles

## Abstract

PI(4,5)P_2_ localizes to sites of dense core vesicle exocytosis in neuroendocrine cells and is required for Ca^2+^-triggered vesicle exocytosis, but the impact of local PI(4,5)P_2_ hydrolysis on exocytosis is poorly understood. Previously, we reported that Ca^2+^-dependent activation of phospholipase Cη2 (PLCη2) catalyzes PI(4,5)P_2_ hydrolysis, which affected vesicle exocytosis by regulating the activities of the lipid-dependent priming factors CAPS (also known as CADPS) and ubiquitous Munc13-2 in PC12 cells. Here we describe an additional role for PLCη2 in vesicle exocytosis as a Ca^2+^-dependent regulator of the actin cytoskeleton. Depolarization of neuroendocrine PC12 cells with 56 or 95 mm KCl buffers increased peak Ca^2+^ levels to ∼400 or ∼800 nm, respectively, but elicited similar numbers of vesicle exocytic events. However, 56 mm K^+^ preferentially elicited the exocytosis of plasma membrane-resident vesicles, whereas 95 mm K^+^ preferentially elicited the exocytosis of cytoplasmic vesicles arriving during stimulation. Depolarization with 95 mm K^+^ but not with 56 mm K^+^ activated PLCη2 to catalyze PI(4,5)P_2_ hydrolysis. The decrease in PI(4,5)P_2_ promoted F-actin disassembly, which increased exocytosis of newly arriving vesicles. Consistent with its role as a Ca^2+^-dependent regulator of the cortical actin cytoskeleton, PLCη2 localized with F-actin filaments. The results highlight the importance of PI(4,5)P_2_ for coordinating cytoskeletal dynamics with vesicle exocytosis and reveal a new role for PLCη2 as a Ca^2+^-dependent regulator of F-actin dynamics and vesicle trafficking.

## Teaser

**Background:** Ca^2+^ and PI(4,5)P_2_ regulate F-actin and vesicle exocytosis in neuroendocrine cells.

**Results:** Phospholipase Cη2 knockdown inhibits Ca^2+^-stimulated PI(4,5)P_2_ hydrolysis, F-actin disassembly, and vesicle recruitment in PC12 cells.

**Conclusion:** Phospholipase Cη2, which localizes with actin, links Ca^2+^ rises to F-actin disassembly and vesicle trafficking.

**Significance:** The results reveal a new role for phospholipase Cη2 as a Ca^2+^-dependent regulator of actin cytoskeletal dynamics and vesicle trafficking.

## Introduction

Peptide secretion from neural and endocrine cells occurs by Ca^2+^-dependent dense core vesicle exocytosis. Vesicles proceed through sequential steps involving transport to the cell periphery followed by plasma membrane tethering, docking, priming, and Ca^2+^-dependent fusion. Essential proteins and lipids function at each step in the regulated secretory pathway (1, 2). PI(4,5)P_2_^2^ is required at a reversible ATP-dependent vesicle priming step (3–5) but regulates other late steps as well. The binding of PI(4,5)P_2_ to its protein effectors is mediated through interactions with pleckstrin homology (PH) and C2 domains or with highly basic motifs in proteins (6–9). PI(4,5)P_2_-binding proteins that function at distinct steps of the exocytic pathway include PH domain-containing CAPS (Ca^2+^-dependent activator protein in secretion, also known as CADPS) (6–9), C2 domain-containing Munc13-1/2 (mammalian homologue of Unc-13-1/2) (9, 10), rabphilin (11), and synaptotagmin-1 (12, 13) and basic motif-containing syntaxin-1 (8, 14).

PI(4,5)P_2_ hydrolysis through receptor-regulated PLCβ or PLCγ is an upstream initiating signal for vesicle exocytosis in neuroendocrine cells by generating inositol 1,4,5-trisphosphate for Ca^2+^ mobilization (15) and DAG to potentiate exocytosis (16, 17). However, there is also a major role for PI(4,5)P_2_ downstream of Ca^2+^ signaling involving distinct pools of PI(4,5)P_2_ (3, 9). High concentration plasma membrane domains of PI(4,5)P_2_ localize near docked vesicles (8, 18, 19) and locally regulate CAPS and Munc13 protein function (9). The Ca^2+^-dependent generation of DAG from PI(4,5)P_2_ potentiates vesicle exocytosis, but Ca^2+^-activated PLCs that are directly linked to vesicle exocytosis had not been identified (20). Several PLCs (*e.g.* PLCδ or PLCη) could function as downstream effectors in Ca^2+^ signaling because of their strong Ca^2+^-dependent activation (21). PLCη2 was recently found to be a Ca^2+^-dependent modulator for CAPS and Munc13 function in neuroendocrine PC12 cells (9).

PI(4,5)P_2_ plays a key role in F-actin assembly mechanisms (22–24). The actin cytoskeleton undergoes dynamic reorganization associated with the Ca^2+^-dependent activation of vesicle exocytosis in secretory cells. F-actin functions in part as a physical barrier in secretory cells to limit vesicle access to the plasma membrane for fusion (25, 26). This actin barrier is locally disassembled during Ca^2+^ rises in stimulated chromaffin cells involving actin-severing proteins such as scinderin (27, 28). In other cell types, PI(4,5)P_2_ hydrolysis catalyzed by PLCγ, PLCβ, or 5-phosphatase has been shown to promote F-actin disassembly (29–32). However, a Ca^2+^-dependent PLC pathway for PI(4,5)P_2_ hydrolysis that reorganizes the actin cytoskeleton in neuroendocrine cells has not been identified.

Neuroendocrine cells possess a plasma membrane-resident pool of vesicles that undergo exocytosis in response to Ca^2+^ rises. Cytoplasmic vesicles are also recruited to the plasma membrane for exocytosis during stimulation (33, 34). We found that varying Ca^2+^ influx in PC12 cells markedly affected whether resident or recruited vesicles undergo exocytosis. Stronger depolarization stimulated more Ca^2+^ entry that uniquely promoted PI(4,5)P_2_ hydrolysis and F-actin disassembly, which in turn enhanced exocytosis of cytoplasmic vesicles arriving during stimulation. PLCη2 was the critical link between increased Ca^2+^ and PI(4,5)P_2_ hydrolysis, F-actin disassembly, and redirected vesicle exocytosis. These studies reveal a functional role for PLCη2 as a Ca^2+^-dependent regulator of the actin cytoskeleton and the secretory pathway in neuroendocrine cells.

## Experimental Procedures

### 

#### 

##### DNA Constructs

The plasmid encoding a green fluorescence protein-tagged BDNF (BDNF-EGFP) was provided by V. Lessmann (Johannes Gutenberg Universität, Mainz, Germany). PKCδ-C1-EGFP (C1-EGFP) was provided by S. Grinstein (Hospital for Sick Children, Toronto, Canada). EGFP-mouse PLCδ1 (EGFP-PLCδ1) and EGFP-mouse PLCη2 (EGFP-PLCη2) were provided by K. Fukami (Tokyo University of Pharmacy and Life Science). To generate PKCδ-C1-mKate2 (C1-mKate2), the PKCδ-C1 domain was amplified from PKCδ-C1-EGFP by PCR using the forward primer 5′-GGACTCAGATCTACCATGGGGG-3′ and reverse primer 5′-ATGTCGACTGGTACCTTGCGCCGGC-3′. The PCR product was digested with BglII and SalI and inserted into BglII and SalI sites of mKate2-N vector. To generate EGFP-PLCη2-PH, the PLCη2-PH domain was amplified from EGFP-PLCη2 by PCR using the forward primer 5′-CTCAGATCTATGCCTGGTCCCCAGCC-3′ and the reverse primer 5′-GCGGTCGACGATGCCAGCCATGAGG-3′. The PCR product was digested with BglII and SalI and inserted into BglII and SalI sites of EGFP-C1 vector. EGFP-PLCη2 3M rescue plasmid was generated by inducing three nonsense mutations in the shRNA targeting sequence by using the forward primer 5′-CGAGCCCTCTCCGATCTCGTGAAATATACC-3′ and the reverse primer 5′-GGTATATTTCACGAGATCGGAGAGGGCTCG-3′.

##### Antibodies and Reagents

Anti-mouse PLCη2 polyclonal antibody was kindly provided by K. Fukami, anti-PLCδ1 (D-7) mouse monoclonal antibody was purchased from Santa Cruz Biotechnology, Inc. (Dallas, TX), and anti-GAPDH monoclonal antibody was purchased from Ambion (Austin, TX). Fluo-4 AM and Alexa Fluor 568 phalloidin were purchased from Molecular Probes, Inc. (Eugene, OR). Other materials and chemicals were obtained from commercial sources.

##### Cell Culture and Transfection

PC12 cells were cultured in Dulbecco's modified Eagle's medium (Sigma) supplemented with 5% horse serum and 5% calf serum at 37 °C in an air plus 10% CO_2_ atmosphere at constant humidity. Transfections for plasmid DNAs were performed by electroporation using an ECM 830 system (BTX, Holliston, MA) set at 230 V, 8 ms, and 1 pulse. PC12 cells (grown to ∼80% confluence in a 10-cm dish) suspended in 0.5 ml of cytomix buffer (25 mm HEPES, 120 mm KCl, 10 mm KH_2_PO_4_, 0.15 mm CaCl_2_, 5 mm MgCl_2_, 2 mm EGTA, pH 7.6) were transfected with 10–50 μg of plasmid DNA(s) using a 4-mm gap size cuvette. Transfections for siRNAs were performed by electroporation using an ECM830 set at 90 V, 8 ms, and 1 pulse. PC12 cells were transfected with 1.33 μm siRNA and 2.5 μg of plasmid DNA using a 1-mm gap size cuvette.

##### Monitoring of DAG Generation on the Plasma Membrane

PC12 cells were transfected with 40 μg of C1-mKate2 plasmid DNA or co-transfected with 25 μg of C1-mKate2 and 25 μg of EGFP, EGFP-PLCδ1, or EGFP-PLCη2 plasmid DNAs and plated on poly-d-lysine-coated (Sigma) and type I collagen-coated (BD Biosciences) 35-mm glass bottom dishes (MatTek Corp., Ashland, MA). After a 48-h incubation, the culture medium was replaced with basal buffer (15 mm HEPES, pH 7.4, 145 mm NaCl, 5.6 mm KCl, 2.2 mm CaCl_2_, 0.5 mm MgCl_2_, 5.6 mm glucose, 0.5 mm ascorbic acid, 0.1% BSA), and then cells were stimulated with 56 (moderate stimulation; MS) and 95 mm K^+^ (strong stimulation; SS) depolarization buffer (basal buffer adjusted to 95 mm NaCl and 56 mm KCl or 56 mm NaCl and 95 mm KCl). Cells were imaged on a Nikon total internal reflection fluorescence (TIRF) microscope evanescent wave imaging system used with a TE2000-U inverted microscope (Nikon) and an Apo TIRF ×100, numerical aperture 1.45 (Nikon) objective lens. EGFP and mKate2 fluorescence were excited with the 488-nm laser line and the 514-nm laser line, respectively. Images were acquired at 250-ms intervals with a CoolSNAP-ES digital monochrome CCD camera system (Photometrics, Tucson, AZ) controlled by Metamorph software (Universal Imaging Corp., Downingtown, PA). All data analysis was conducted with ImageJ software.

##### TIRF Analysis of BDNF-EGFP Secretion

PC12 cells were transfected with 30 μg of BDNF-EGFP plasmid DNA and plated on poly-d-lysine- and collagen-coated 35-mm glass bottom dishes. After a 48-h incubation, the culture medium was replaced with basal buffer, and cells were stimulated with MS or SS buffer. Cells were imaged on the Nikon TIRF microscope at 250-ms intervals with a CoolSNAP-ES digital monochrome CCD camera system (Photometrics) controlled by Metamorph software (Universal Imaging Corp.). The penetration depth (1/*e*) of the evanescent field was estimated to be 160 nm based on a calibration with fluorescent beads. At this penetration depth, resident vesicles (estimated *d* = 100 nm) at the plasma membrane are evident, whereas vesicles enmeshed deeper in the 400-nm actin cortex (34), termed non-resident cytoplasmic vesicles, are dim or not evident. Exocytic events were manually counted and scored for whether exocytosis occurred from vesicles resident in the evanescent field for ≥0.5 s (resident) or <0.5 s (non-resident) prior to fusion. All data analysis used Metamorph software.

##### Ca^2+^ Imaging

PC12 cells were plated on poly-d-lysine- and collagen-coated 35-mm glass bottom dishes. After a 24-h incubation, cells were washed, and the culture medium was replaced with basal buffer. Cells were loaded with fluo-4 by incubation with 2 μm fluo-4, AM and 0.02% Pluronic® F-127 (Molecular Probes) mixture at room temperature for 30 min in the dark. Cells were then washed with basal buffer and incubated at 37 °C for 20 min to allow de-esterification of loaded dye in basal buffer. Cells were stimulated with MS or SS buffer, and images were acquired at 250-ms intervals on an epifluorescence microscope (Nikon). Cells were treated with 5 μm ionomycin and 5 mm EGTA to obtain maximum (*f*_max_) and minimum (*f*_min_) fluorescence values, respectively. Average fluorescence intensity at each time point (*f_t_*) was measured using Metamorph software. Relative fluorescence intensity of fluo-4-Ca^2+^ (*F*) and concentration of intracellular Ca^2+^ ([Ca^2+^]*_i_*) were determined as *F* = (*f_t_* − *f*_min_)/(*f*_max_ − *f*_min_) and [Ca^2+^]*_i_* = *K_D_ F*/(1 − *F*), where *K_D_* for fluo-4 is 345 nm.

##### Knockdown of PLCη2 by shRNA and siRNA

PC12 cells were co-transfected with 30 μg of pSM2-PLCη2 shRNA vector; V2MM_89060 (shRNA 1) and V2MM_197066 (shRNA 2) targeting mouse PLCη2 mRNA (accession number NM_001113360) sequence corresponding to nucleotides 2117–2135 (CCCTCTCGGACCTAGTGAA) and 2119–2137 (CTCTCGGACCTAGTGAAAT), respectively (Open Biosystems, Huntsville, AL); or pSM2 empty vector (Open Biosystems) and 10 μg of C1-mKate2 plasmid DNAs and plated on poly-d-lysine- and collagen-coated 35-mm glass bottom dishes for TIRF analysis and 6-well dishes for Western blotting. After a 72-h incubation, cells were lysed and subjected to blotting analysis with anti-PLCη2 polyclonal antibody and anti-GAPDH monoclonal antibody. For rescue experiments, PC12 cells were triple-transfected with 30 μg of pSM2-PLCη2 shRNA or pSM2 empty vector, 10 μg of C1-mKate2 plasmid DNA, and 5 μg of EGFP-PLCη2 3M plasmid DNA. Monitoring of DAG generation was performed as described above. PC12 cells were co-transfected with 35 μg of pSM2-PLCη2 shRNA or pSM2 empty vector and 15 μg of BDNF-EGFP plasmid DNAs and plated on poly-d-lysine- and collagen-coated 35-mm glass bottom dishes for TIRF analysis and a 6-well dish for blotting. After a 72-h incubation, blotting analysis and TIRF analysis were performed as described above. Knockdown of PLCη2 by endoribonuclease-prepared siRNA (target sequence corresponds to nucleotides 1406–1942 of mRNA isolated from PC12 cells) was performed as described previously (9).

##### Co-localization Analysis

Co-localization analysis of signals corresponding to PLCη2 and F-actin was performed using a pixel by pixel analysis algorithm with Fiji-win32 software. The calculated percentage of random co-localization was subtracted from co-localization values.

##### Quantification of Cortical F-actin

EGFP-, EGFP-PLCδ1-, and EGFP-PLCη2-overexpressing and PLCη2 knockdown (EGFP co-transfection marker indicated that transfection efficiency was 82%) PC12 cells were plated on poly-d-lysine- and collagen-coated 35-mm glass bottom dishes. After a 48-h (or 72-h for PLCη2 knockdown) incubation, cells were treated with MS or SS buffers at room temperature for 0.5, 1, 2, and 3 min. After treatment, the cells were immediately fixed with 3.7% formaldehyde in PBS at room temperature for 8 min and permeabilized by incubation with 0.1% Triton X-100 in PBS containing 1% BSA in PBS at room temperature for 10 min. F-actin was visualized with Alexa Fluor 568-phalloidin at room temperature for 20 min followed by washing. Cells were imaged on a TIRF microscope with a CoolSNAP-ES digital monochrome CCD camera system controlled by Metamorph software, and data analysis utilized Metamorph software.

## Results

### 

#### 

##### Strong Stimulation Promotes Greater Ca^2+^ Rises and PI(4,5)P_2_ Hydrolysis

**FIGURE 1.**
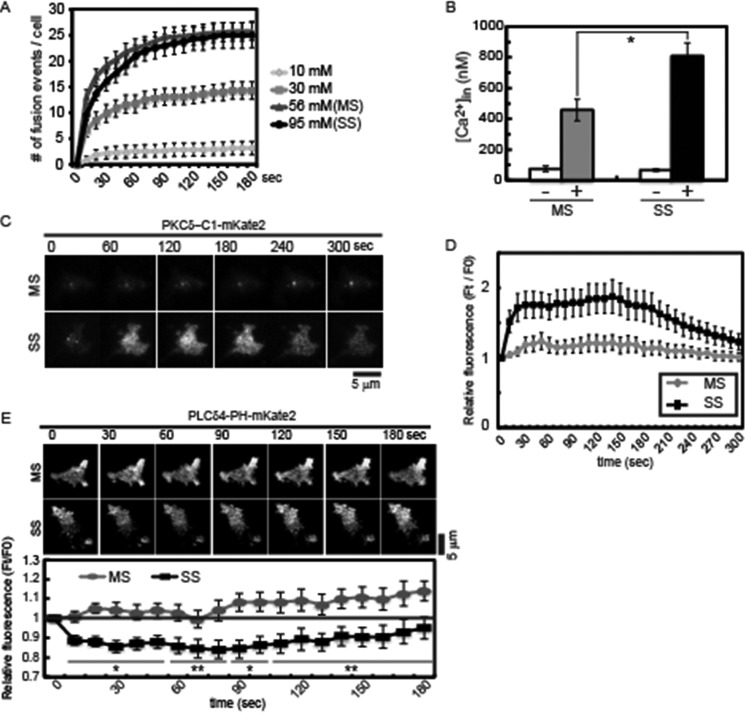
**Depolarization in 95 mm KCl elicits greater Ca^2+^ influx and DAG generation.** *A*, depolarization-dependent increases in the number of BDNF-EGFP fusion events. PC12 cells expressing BDNF-EGFP were stimulated with the indicated KCl-containing buffers with 2.2 mm CaCl_2_. Images were acquired by TIRF microscopy at 4 Hz, and fusion events were identified and shown as cumulative plots (mean ± S.E. (*error bars*), *n* = 10 cells). *B*, peak intracellular [Ca^2+^] at 5–10 s in PC12 cells loaded with fluo-4 was determined for cells incubated in 56 mm KCl (MS) (*n* = 9 cells) and 95 mm KCl (SS) (*n* = 10 cells) buffers. Mean values ± S.E. are shown (*, *p* < 0.005). *C*, DAG generation under MS and SS conditions was determined by monitoring PKCδ-C1-mKate2 translocation to the plasma membrane by TIRF microscopy at 4 Hz. *D*, relative fluorescence intensities of images from *C* were plotted (mean ± S.E., *n* = 10–14 cells). *E*, plasma membrane PI(4,5)P_2_ levels in PC12 cells incubated in MS and SS buffers. Cells expressing PLCδ4-PH-mKate2 were incubated in MS or SS buffers for the times indicated and imaged by TIRF microscopy at 4 Hz. Representative images (*top*) indicate a transient decrease in PI(4,5)P_2_ levels in cells incubated with SS but not MS buffers. The relative fluorescence of cell footprints was quantitated at 10-s intervals (mean ± S.E., *n* = 8 cells; *, *p* < 0.005; **, *p* < 0.05). The PI(4,5)P_2_ decrease in SS buffers was partial.

PI(4,5)P_2_ is required for regulated vesicle exocytosis and is distributed in membrane domains present at sites of exocytosis (8, 9, 19). To determine the impact of PI(4,5)P_2_ hydrolysis on regulated vesicle exocytosis, we utilized a range of stimulation conditions in the well characterized PC12 cell model for neuroendocrine secretion. TIRF microscopy was used to monitor the exocytosis of vesicles containing fluorescent cargo proteins (35, 36) and to detect PI(4,5)P_2_ hydrolysis and DAG generation (9, 37). Depolarizing the cells by incubation in high KCl buffers promotes depolarization and Ca^2+^ influx. PC12 cells have a low density of L-type Ca^2+^ channels so that substantial depolarization in KCl buffers is required to elicit Ca^2+^ increases (38, 39). We found that 56 mm KCl (MS) was optimal for promoting maximal number of dense core vesicle fusion events of BDNF-EGFP within 3 min (Fig. 1*A*). Stronger depolarization at 95 mm KCl (SS) did not further increase the number of fusion events (Figs. 1*A* and 3*C*). Analysis of the time courses of averaged cumulative fusion events that fit well to an exponential function indicated that the time course of SS-evoked fusion events tended to be slower than MS-evoked fusion events (time constant τ values for MS and SS were 19.7 ± 2.3 s and 29.5 ± 4.9 s, respectively, *p* = 0.09). MS conditions promoted a peak rise in [Ca^2+^] to ∼400 nm, whereas stronger depolarization with SS conditions evoked a peak [Ca^2+^] rise to ∼800 nm (Fig. 1*B*), similar to a previous report (39). The results indicate that Ca^2+^-dependent vesicle exocytosis is saturated by the Ca^2+^ concentration rises elicited by MS conditions. In subsequent studies, we utilized MS and SS conditions to promote optimal (∼400 nm) or greater than optimal (∼800 nm) Ca^2+^ rises, respectively.

Under the MS and SS buffer stimulation conditions, lipid signaling events differed markedly. A fluorescent PKCδ-C1-mKate2 probe was used to detect DAG generation in the plasma membrane by TIRF microscopy (37). Under MS conditions, where vesicle exocytosis was maximally stimulated (Fig. 1*A*), plasma membrane DAG levels did not differ from those in unstimulated cells (Fig. 1, *C* and *D*). By contrast, SS conditions resulted in a rapid (∼15 s), transient increase in DAG levels, as inferred from the translocation of the C1-mKate2 protein (Fig. 1, *C* and *D*). The translocated C1-mKate2 was evident as bright puncta and diffuse fluorescence (Fig. 1*C*), which suggested that high concentration domains of DAG may be generated from high concentration domains of PI(4,5)P_2_ (9) followed by diffusion. Using a PLCδ4-PH-mKate2 domain probe with TIRF (9), we detected a corresponding partial loss of PI(4,5)P_2_ from the plasma membrane under SS but not under MS buffer conditions (Fig. 1*E*). The results indicate that PI(4,5)P_2_ hydrolysis with DAG generation was only promoted at Ca^2+^ concentrations higher (SS conditions) than those needed to maximally stimulate vesicle exocytosis (MS conditions).

##### PLCη2 Mediates PI(4,5)P_2_ Hydrolysis at Elevated Ca^2+^ Levels

**FIGURE 2.**
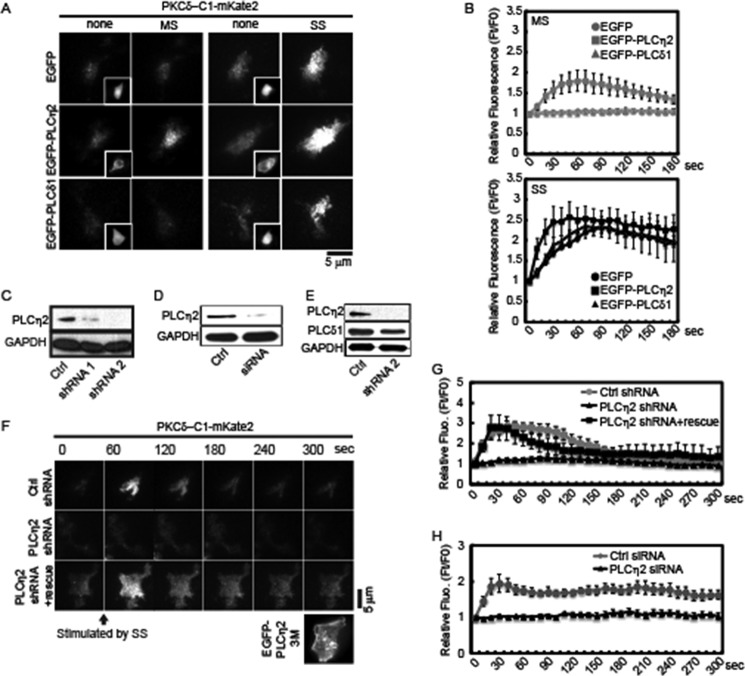
**PLCη2 generates DAG at the plasma membrane in response to high K^+^-induced depolarization.** *A*, effect of PLC overexpression on DAG generation under resting or MS and SS conditions. EGFP, EGFP-PLCη2, and EGFP-PLCδ1 were co-expressed with PKCδ-C1-mKate2 in PC12 cells. After 48 h, the cells were incubated in resting conditions or in MS or SS buffers, and images were acquired at 4 Hz by TIRF microscopy. Images show the point of maximal fluorescence increase with *insets* showing the distribution of EGFP and EGFP fusion proteins before incubation by epifluorescence microscopy. *B*, averages of relative fluorescence intensity of PKCδ-C1-mKate2 in cell footprints during incubation in MS (*top*) or SS buffer (*bottom*) from TIRF images similar to *A* are plotted (*n* = 9 cells). *C* and *D*, PLCη2 levels were determined in PC12 cells transfected with a control shRNA plasmid (*C*) or siRNA (*D*) or with either of two PLCη2 shRNA plasmids (*C*) or PLCη2 siRNA (*D*). *E*, expression levels of PLCδ1 in PLCη2 KD PC12 cells. PLCδ1 was detected by Western blotting with anti-PLCδ1 antibody in cells treated with PLCη2 shRNA 2. *F*, PLCη2 is required for DAG generation elicited under SS conditions. PC12 cells were co-transfected with PLCη2 shRNA 2 (*n* = 12 cells) or with empty vector (*n* = 10 cells) along with an expression plasmid for PKCδ-C1-mKate2. For rescue studies, PC12 cells were triple-transfected with PLCη2 shRNA 2, PKCδ1-C1-mKate2, and EGFP-PLCη2 3M plasmid (*n* = 12 cells). Expressed EGFP-PLCη2 3M is shown in the *inset*. After 72 h, cells were incubated in SS buffer for the indicated times. Images were acquired at 4 Hz by TIRF microscopy with representative frames shown. *G* and *H*, the relative fluorescence intensity of PKCδ-C1-mKate2 in cell footprints during incubation is shown (mean ± S.E. (*error bars*), *n* = 10–12 cells).

The greater Ca^2+^ elevation under SS conditions probably stimulated DAG generation from the Ca^2+^-dependent activation of a PI(4,5)P_2_-hydrolyzing PLC. Because PLCδ1 and PLCη2 are expressed in PC12 cells (data not shown) (40) and are strongly activated by Ca^2+^ (21), we determined which if either was responsible for DAG generation in response to Ca^2+^ elevations under SS conditions. We first determined the effect of PLCδ1 and PLCη2 overexpression on DAG generation. A PKCδ-C1-mKate2 probe that monitored DAG was translocated to the plasma membrane in EGFP-expressing control cells under SS conditions but not under MS conditions (Fig. 2, *A* (*top*) and *B*), whereas expression of a EGFP-PLCη2 protein enhanced DAG generation even under MS conditions (Fig. 2, *A* (*middle*) and *B*). This contrasted with cells expressing EGFP-PLCδ1 (Fig. 2, *A* (*bottom panels*) and *B*), where there was no DAG generation beyond that of control cells. These findings indicate that PLCη2 rather than PLCδ1 responds to Ca^2+^ influx by generating DAG in PC12 cells, which was consistent with *in vitro* studies indicating the greater Ca^2+^ sensitivity for PLCη2 activation compared with PLCδ1 (41).

To determine whether endogenous PLCη2 was responsible for DAG generation in control cells under SS conditions, we utilized shRNA plasmids and siRNA that were effective in reducing PLCη2 by more than 90 and 80%, respectively (Fig. 2, *C* and *D*). Depletion of PLCη2 did not affect the expression level of PLCδ1 (Fig. 2*E*). In cells depleted of PLCη2 by either shRNA plasmid or siRNA, DAG generation in response to SS buffer was completely abolished (Fig. 2, *F–H*). Both shRNA plasmids, but not a control plasmid, had similar effects (data not shown). To confirm that the loss of DAG generation in PLCη2-depleted cells was due to the lack of PLCη2, we conducted rescue experiments with an shRNA-resistant EGFP-PLCη2 construct (EGFP-PLCη2 3M) that contained three nucleotide substitutions in the shRNA target. Expression of EGFP-PLCη2 3M restored DAG generation (Fig. 2, *F* and *G*). These findings indicate that PLCη2 is the major PLC in PC12 cells that is activated at the plasma membrane by the higher Ca^2+^ concentrations promoted by strong depolarization under SS conditions.

##### Strong Stimulation Shifts Exocytosis from Docked to Newly Arrived Vesicles

**FIGURE 3.**
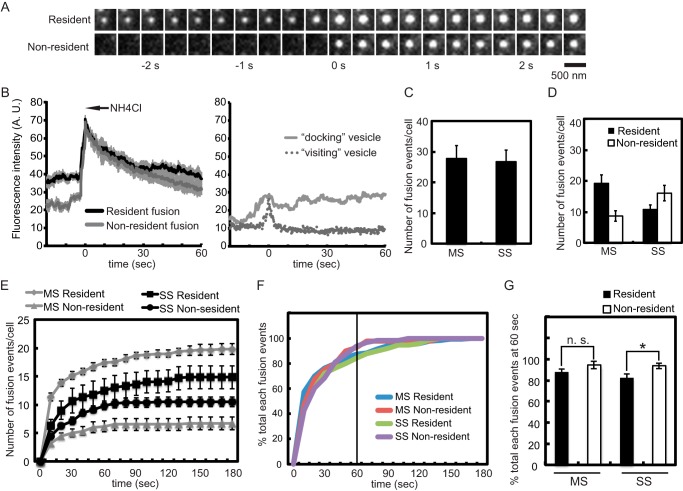
**Vesicle exocytosis shifts from resident to non-resident vesicles under SS conditions.** *A*, montages showing resident (*top*) and non-resident (*bottom*) vesicle exocytic events imaged at 4 Hz by TIRF microscopy. BDNF-EGFP-containing vesicles brighten at the point of fusion pore formation (termed 0 s) due to pH change. The BDNF-EGFP subsequently dims due to fusion pore closure and vesicle re-acidification. Non-resident vesicles are not visible in the TIRF field for at least 0.5 s prior to fusion. *B*, fluorescence changes in fusing and non-fusing vesicles. *Left*, average relative fluorescence of individual vesicles corresponding to residents or non-residents that underwent exocytosis in response to stimulation (*n* = 10 events). Profiles are similar with non-residents starting at lower background values. An *arrow* indicates the average relative fluorescence change with 50 mm NH_4_Cl-induced vesicle pH neutralization (*n* = 10 vesicles). *Right*, relative fluorescence of individual vesicles that failed to fuse in stimulated cells corresponding to vesicles that briefly (*visiting*) or stably (*docked*) entered the TIRF field. *C* and *D*, PC12 cells expressing BDNF-EGFP were incubated in MS (*n* = 9 cells) and SS (*n* = 11 cells) buffers for 5 min. Images of exocytic events were acquired at 4 Hz by TIRF microscopy. Mean values ± S.E. (*error bars*) (*n* = 9–11 cells) are plotted. *C*, the total number of exocytic events per cell elicited by MS or SS buffers is shown. *D*, exocytic events were counted and categorized into resident and non-resident events. *E*, time course of each type of exocytic event. The numbers of each type of exocytic event (same data set as in Fig. 1*A*) under MS and SS conditions are presented as a cumulative plot. *F* and *G*, cumulative distribution of each type of exocytic event (*F*). Within 60 s, 88, 94, 83, and 94% of MS-resident, MS-non-resident, SS-resident, and SS-non-resident types of exocytic events occurred, respectively (*G*) (*, *p* < 0.05; *n.s.*, not significant). *A.U.*, arbitrary units.

PI(4,5)P_2_ is required for dense core vesicle exocytosis (3, 18, 42). However, the SS conditions that promoted PI(4,5)P_2_ hydrolysis by PLCη2 did not reduce the total number of exocytic events in 5 min (Fig. 3*C*). This is accounted for by the fact that PI(4,5)P_2_ hydrolysis is partial under SS stimulation conditions (Fig. 1*E*) and that the DAG generated (Fig. 1*D*) further activates ubMunc13-2 (9). To determine more fully the impact of PI(4,5)P_2_ hydrolysis on Ca^2+^-triggered vesicle exocytosis, we examined individual exocytic events by TIRF microscopy. As previously characterized for PC12 cells (34, 36, 43), two types of evoked vesicle exocytic events were observed: from resident vesicles present at the plasma membrane for ≥0.5 s before fusion or from non-resident cytoplasmic vesicles that arrived <0.5 s before fusion in stimulated cells (Fig. 3*A*). The vesicle exocytosis assay utilized in TIRF studies employing BDNF-EGFP cargo has been extensively characterized (35). Resident and non-resident vesicles that fuse are readily distinguished from non-fusing, vesicles as shown by the traces of fluorescent changes in Fig. 3*B*. Fusing vesicles exhibit hallmark features of an initial brightening upon fusion pore formation (due to vesicle pH change) followed by slow dimming as the fusion pore closes and the vesicles re-acidify in cavicapture exocytosis (Fig. 3*B*, *left*). In accord with this, a similar brightening was obtained by neutralizing vesicle pH by treatment with 50 mm NH_4_Cl (Fig. 3*B*, *left*, *arrow*). The initial fluorescence of resident vesicles prior to fusion is greater than that for non-resident vesicles, which is at background (Fig. 3, *A* and *B* (*left*)). By contrast, non-fusing vesicles that approach the plasma membrane and dock or approach the plasma membrane and leave lack a fusion spike (Fig. 3*B*, *right*). Such vesicles are rare in stimulated cells and are readily distinguished from fusing vesicles. Under MS stimulation conditions, ∼70% of exocytic events occurred from resident vesicles and ∼30% from non-resident vesicles (Fig. 3*D*), similar to previous studies (36, 43). By contrast, under SS stimulation conditions, the number of exocytic events from resident vesicles decreased, and those from non-resident vesicles increased (Fig. 3*D*), resulting in ∼40% of exocytic events from resident vesicles and ∼60% from newly arrived non-resident vesicles. We analyzed the cumulative time courses for each type of fusion event evoked under MS and SS conditions. In both MS and SS conditions, the time course of resident *versus* non-resident vesicle fusion events did not differ significantly (τ value for MS-resident *versus* non-resident: 16.1 ± 1.5 *versus* 19.8 ± 2.5 s; for SS resident *versus* non-resident: 26.93 ± 3.5 *versus* 21.8 ± 2.7 s) (Fig. 3*E*). However, the time course of resident vesicle fusion events under SS conditions (τ = 26.93 ± 3.5 s) was significantly (*p* < 0.05, *n* = 10) slower than that under MS condition (τ = 16.1 ± 1.5 s). Increased Ca^2+^ influx under SS conditions favored the fusion of newly arriving vesicles and reduced the number of and slowed down resident vesicle fusion events. Most fusion events occurred within 1 min after stimulation under both MS and SS conditions (Fig. 3, *F* and *G*).

##### PLCη2 Activation Switches Exocytosis from Docked to Newly Arrived Vesicles

**FIGURE 4.**
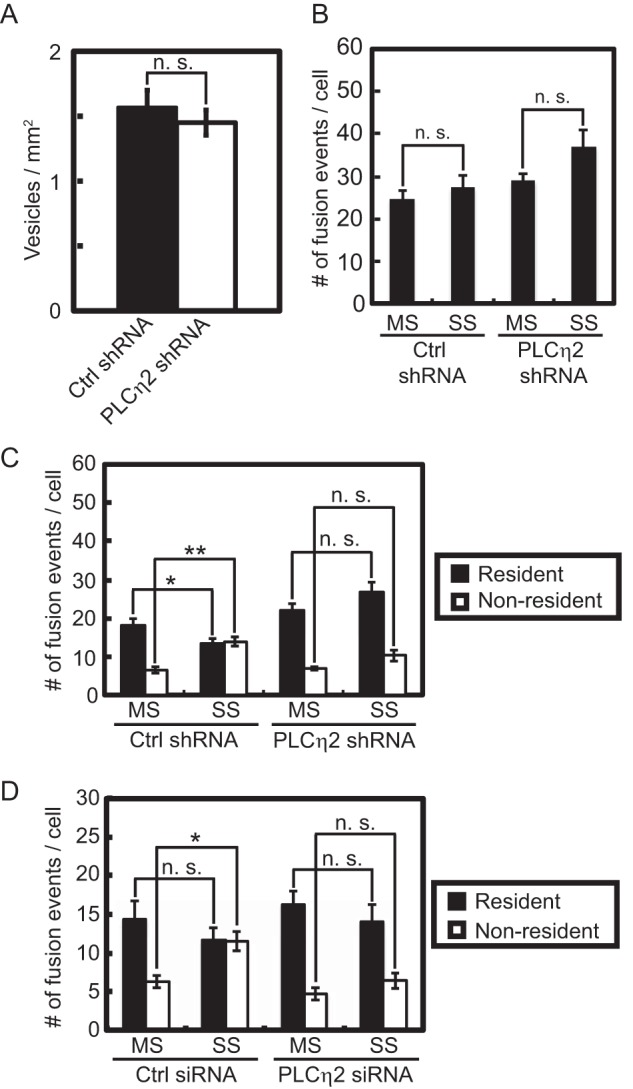
**Knockdown of PLCη2 affects the shift in exocytosis from resident to non-resident vesicles.** Cells were co-transfected with BDNF-EGFP and shRNA plasmid targeting PLCη2 (*n* = 12 cells) or empty vector (*n* = 10 cells). *A*, bar graphs show the average density of vesicles in the TIRF field for control and PLCη2 knockdown cells (mean ± S.E. (*error bars*)). *B* and *D*, PLCη2 knockdown or control cells were incubated in MS (*n* = 7–8 cells) and SS (*n* = 7–8 cells) buffers. The total exocytic events for 5 min were counted (*B*), categorized into resident and non-resident events, and expressed as number/cell (*C*). *D*, PLCη2 knockdown by siRNA or control cells were incubated in MS (*n* = 8 control cells, *n* = 13 KD cells) and SS (*n* = 8 control cells, *n* = 9 KD cells) buffers. The total exocytic events for 5 min were counted and categorized into resident and non-resident events and expressed as number/cell (mean ± S.E.; *, *p* < 0.05; **, *p* < 0.001; *n.s.*, not significant).

Because PLCη2 activation was restricted to SS stimulation conditions, we determined whether PLCη2 was essential for switching the pathway for vesicle exocytosis from resident to non-resident vesicles. PLCη2 knockdown did not affect the density of resident vesicles evident in the evanescent field (Fig. 4*A*). In both control and PLCη2 knockdown cells, the number of total exocytic events was similar under MS and SS conditions (Fig. 4*B*, *bar 1 versus bar 2* and *bar 3 versus bar 4*), although there was a trend for PLCη2 knockdown to increase the total number of exocytic events under SS conditions (Fig. 4*B*, *bar 1 versus bar 3* and *bar 2 versus bar 4*; not significant), as expected for increased PI(4,5)P_2_ levels in the cells (18, 19, 44). The pathway for vesicle exocytosis shifted from resident to non-resident vesicles under SS stimulation conditions in control cells (Fig. 4*C*). However, in PLCη2 knockdown cells, the shift from resident to non-resident vesicle exocytosis under SS conditions failed to occur (Fig. 4*C*). Similar results were obtained in PLCη2 siRNA knockdown cells (Fig. 4*D*). These results establish that PLCη2 activation is responsible for the shift in the exocytic pathway from resident to non-resident vesicles promoted by greater elevations in Ca^2+^.

##### F-actin Disassembly Is Necessary for the Shift in the Exocytic Pathway

**FIGURE 5.**
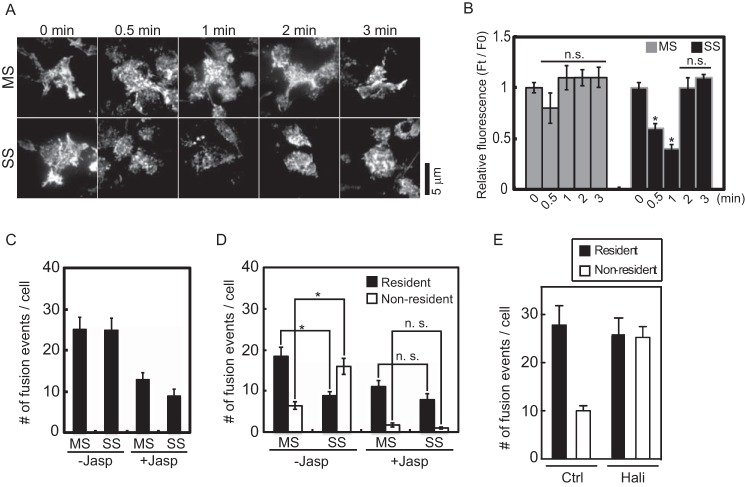
**Stabilization of F-actin prevents whereas drug-induced F-actin disassembly promotes a shift from resident to non-resident vesicle exocytosis.** *A*, effect of incubation buffers on cortical F-actin. PC12 cells were incubated in control or MS or SS buffers for 0.5, 1, 2, and 3 min; fixed; permeabilized; and stained with Alexa Fluor 568 phalloidin for imaging by TIRF microscopy. *B*, the mean fluorescence intensity ± S.E. (*error bars*) of cell footprints was determined (*n* = 15–20 cells; *, *p* < 0.0001; *n.s.*, not significant). *C* and *D*, effect of jasplakinolide (*Jasp*) treatment on resident and non-resident vesicle exocytic events. BDNF-EGFP-expressing cells were treated with 1 μm jasplakinolide at 37 °C for 30 min and incubated in MS or SS buffers for 5 min. The total exocytic events were counted (*C*), categorized into resident and non-resident events, and plotted as number/cell (*D*) (mean ± S.E.; *n* = 10–12 cells; *, *p* < 0.005). *E*, effect of halichondramide (*Hali*) treatment on the evoked exocytosis of resident and non-resident vesicles. The fusion events were counted and categorized into resident and non-resident events. (mean ± S.E., *n* = 6–15 cells).

Cortical F-actin acts as a barrier or cage to restrict vesicle access to the plasma membrane for fusion (25, 26). Because PI(4,5)P_2_ regulates the actin cytoskeleton during vesicle exocytosis (45, 46), we determined whether PLCη2-catalyzed PI(4,5)P_2_ hydrolysis caused disassembly of F-actin, which would increase the access of non-resident vesicles to the plasma membrane. The assembled state of cortical F-actin was assessed by fluorescent phalloidin staining of cells viewed by TIRF microscopy. The assembled state of cortical F-actin did not significantly change after 0.5, 1, 2, or 3 min after application of MS buffer. By contrast, in SS buffer, cortical F-actin strongly decreased by 0.5 min (∼42%) and 1 min (∼62%) (Fig. 5, *A* and *B*) but reassembled by 2–3 min (Fig. 5*B*). The F-actin dynamics correlated with the dynamics of PI(4,5)P_2_ hydrolysis/DAG generation (Fig. 1, *C–E*). Moreover, non-resident vesicle fusion events were completed within this period (Fig. 3, *E–G*). The data suggest that PI(4,5)P_2_ hydrolysis-dependent cortical F-actin disassembly might enable non-resident vesicle fusion events under SS condition.

Pharmacological treatments were used to determine whether F-actin disassembly was necessary or sufficient to shift the exocytic pathway from resident to non-resident vesicles. Treatment with jasplakinolide, an F-actin-stabilizing drug, inhibited fusion events under both MS and SS conditions (Fig. 5*C*, *bar 1 versus bar 3* and *bar 2 versus bar 4*). In control cells (−*Jasp*), the number of exocytic events from resident vesicles was reduced, and that from non-resident vesicles was increased under SS conditions (Fig. 5*D*, *bars 1–4*). Treatment with jasplakinolide (+*Jasp*) mainly suppressed exocytic events from non-resident vesicles, especially under SS conditions (Fig. 5*D*, *bars 5–8*). These data suggested that stabilization of F-actin by jasplakinolide preferentially affected the exocytosis of non-resident vesicles, thereby preventing an increase under SS conditions. Conversely, we promoted F-actin disassembly by treatment with halichondramide (*Hali*), an actin filament-severing and capping drug (47). Under MS conditions, halichondramide treatment increased the number of non-resident vesicle exocytic events without affecting the number of resident events (Fig. 5*E*). Halichondramide treatment mimicked the effect of SS conditions in enhancing non-resident exocytic events under MS condition. These data indicated that F-actin disassembly was sufficient to enhance the exocytosis of non-resident vesicles. Overall, the opposite effects of jasplakinolide and halichondramide treatment were consistent with a role for F-actin disassembly in enabling the fusion of non-resident vesicles.

##### PLCη2 Regulates F-actin Disassembly

**FIGURE 6.**
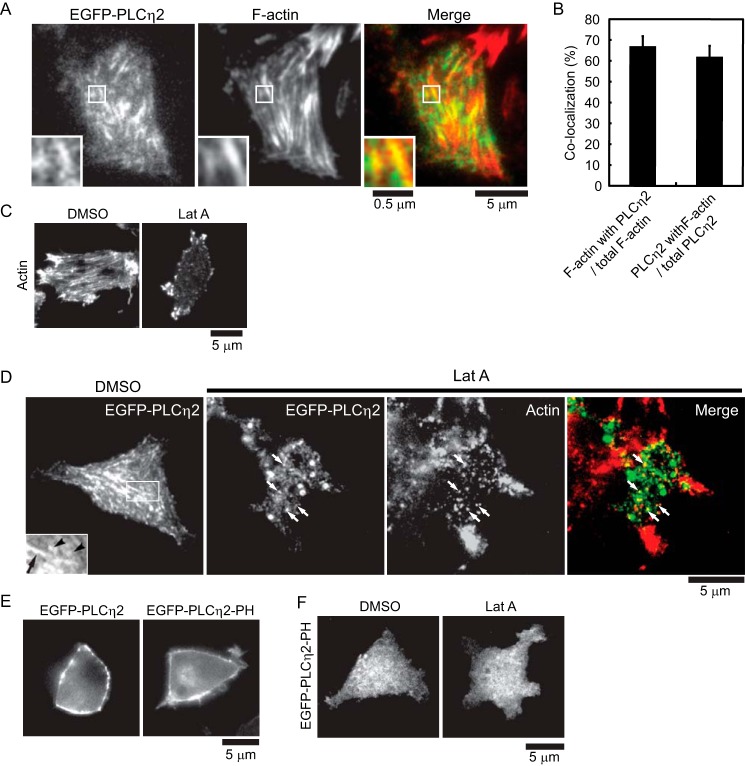
**Co-localization of EGFP-PLCη2 and F-actin in PC12 cells.** *A*, EGFP-PLCη2-expressing cells were fixed, permeabilized, and incubated with Alexa Fluor 568 phalloidin for F-actin imaging by TIRF microscopy. *Insets* show portions of the image (*boxed*) at higher magnification. *B*, the co-localization of EGFP-PLCη2 and phalloidin-stained F-actin was quantitated as described under “Experimental Procedures” (mean ± S.E. (*error bars*), *n* = 12 cells). *C*, effect of latrunculin A (*Lat A*) on cortical F-actin. Cells were incubated with DMSO or with 1 μm latrunculin A at 37 °C for 5 min and fixed, permeabilized, and stained with Alexa Fluor 568 phalloidin for imaging by TIRF microscopy. *D*, effect of latrunculin A treatment on localization of EGFP-PLCη2. EGFP-PLCη2-expressing cells were incubated with DMSO or with 1 μm latrunculin A at 37 °C for 5 min and imaged by TIRF microscopy. In latrunculin A-treated cells, EGFP-PLCη2 distributed to puncta and small structures that were near residual F-actin-containing structures stained with Alexa Fluor 568 phalloidin (*arrows*). *E*, the localization of EGFP-PLCη2 and EGFP-PLCη2-PH by epifluorescence microscopy. *F*, effect of latrunculin A on localization of EGFP-PLCη2-PH domain. EGFP-PLCη2-PH-expressing cells were incubated with DMSO or with 1 μm latrunculin A at 37 °C for 5 min and imaged by TIRF microscopy.

The previous results indicated that PLCη2 activation shifts exocytosis from resident to non-resident vesicles and that F-actin disassembly was in part responsible for the shift. To determine the relationship of PLCη2 to cortical F-actin, co-localization studies were conducted. TIRF microscopy revealed that EGFP-PLCη2 was distributed in punctate and in filamentous structures, with the latter co-localizing with F-actin (Fig. 6, *A* and *B*). Because PLCη2 was reported to localize to the plasma membrane by binding to PI(4,5)P_2_ via its PH domain (41, 48), the observed filamentous distribution of EGFP-PLCη2 (Fig. 6*A*) could result from binding to PI(4,5)P_2_-rich membrane domains that co-localize along F-actin filaments. Alternatively, the filamentous distribution might correspond to direct binding of EGFP-PLCη2 to F-actin or to actin-binding proteins. To distinguish these alternatives, we determined the localization of EGFP-PLCη2 following treatment with latrunculin A to disassemble F-actin. Latrunculin A treatment effectively disassembled F-actin (Fig. 6*C*) and eliminated the filamentous distribution of EGFP-PLCη2 (Fig. 6*D*) detected by TIRF microscopy. Latrunculin treatment produced small structures and puncta of EGFP-PLCη2 with some nearly residual actin-containing structures (Fig. 6*D*, *arrows*). By contrast, the PH domain of PLCη2 (EGFP-PLCη2-PH), which localized to the plasma membrane similarly to full-length EGFP-PLCη2 by epifluorescence (Fig. 6*E*), localized rather differently into broadly distributed small puncta rather than filaments by TIRF microscopy, and this distribution was not altered by latrunculin A treatment (Fig. 6*F*). The results suggest that the filamentous distribution of EGFP-PLCη2 near the plasma membrane is mediated by a direct interaction with F-actin or actin-binding proteins utilizing a domain of PLCη2 other than its PH domain.

**FIGURE 7.**
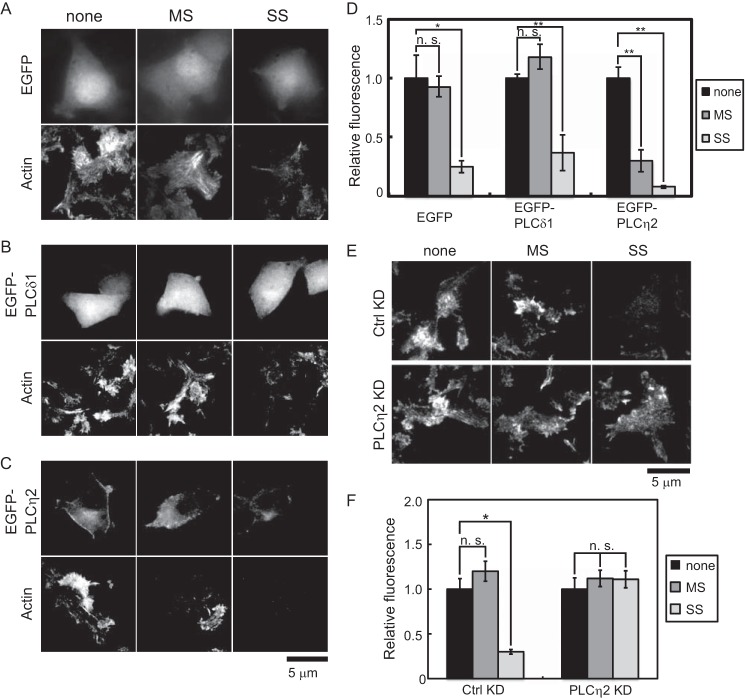
**Overexpression and knockdown of PLCη2 affect F-actin disassembly in PC12 cells.** *A–C*, effect of PLC overexpression on cortical F-actin disassembly under MS and SS conditions. PC12 cells expressing EGFP (*A*), EGFP-PLCδ1 (*B*), and EGFP-PLCη2 (*C*) were incubated in MS and SS buffer for 1 min, fixed, permeabilized, and incubated with Alexa Fluor 568 phalloidin. EGFP proteins were imaged by epifluorescence (*top panels*) and phalloidin (*bottom panels*) by TIRF microscopy. *D*, the mean fluorescence intensity ± S.E. (*error bars*) of Alexa Fluor 568 phalloidin in a footprint was determined (*n* = 6–12 cells; *, *p* < 0.01; **, *p* < 0.0001). *E*, effect of PLCη2 knockdown on cortical F-actin disassembly. Control or PLCη2 knockdown cells were incubated in control, MS, or SS buffers for 1 min and processed for Alexa Fluor 568 phalloidin-binding of F-actin imaged by TIRF microscopy. *F*, mean fluorescence intensity ± S.E. of phalloidin in cell footprints (*n* = 8–10 cells; *, *p* < 0.0005; *n.s.*, not significant).

To further link the activation of PLCη2 to the state of F-actin assembly, we determined the effect of PLC overexpression on F-actin disassembly. In control EGFP-expressing cells, cortical F-actin visualized by fluorescent phalloidin was disassembled when cells were incubated under SS but not MS conditions (Fig. 7, *A* and *D*). Cells expressing EGFP-PLCδ1 exhibited changes very similar to control cells (Fig. 7, *B* and *D*). By contrast, overexpression of EGFP-PLCη2 resulted in the disassembly of cortical F-actin even under MS conditions (Fig. 7, *C* and *D*), which was similar to the results for DAG generation (Fig. 2, *A* and *B*). Last, to confirm that PLCη2 activation affects F-actin disassembly, we determined the impact of PLCη2 knockdown. In control cells, cortical F-actin was disassembled under SS but not MS conditions (Fig. 7, *E* and *F*). By contrast, neither MS nor SS conditions induced cortical F-actin disassembly in PLCη2 knockdown cells (Fig. 7, *E* and *F*). The results indicate that PLCη2 activation promotes F-actin disassembly.

## Discussion

Studies in many cell types have described the important role of PI(4,5)P_2_ in enabling F-actin assembly (24). In addition, multiple roles for F-actin in vesicle trafficking and exocytosis have been characterized for neuroendocrine cells (49). It has been shown that increases in cytoplasmic Ca^2+^ trigger F-actin disassembly (25, 28, 49–52), which increases the access of cytoplasmic recruitment vesicles to the plasma membrane for fusion (33, 34). The current study reveals that PLCη2 is a critical link between Ca^2+^ rises and the disassembly of the F-actin cytoskeleton for regulating vesicle trafficking to the plasma membrane for fusion.

PLCη2 is mainly expressed in neural and endocrine secretory cells, but a functional cellular role for the enzyme has not previously been characterized (41, 53, 54). The strong Ca^2+^ dependence of the activation of PLCη2 suggested that it was a Ca^2+^-dependent effector for unidentified neural/endocrine processes (41). Gβγ subunits also activate PLCη2, which could indicate receptor-regulated roles for this enzyme as well (55). However, PLCη2 knock-out mice did not exhibit obvious phenotypes that would suggest a functional role (56). By contrast, the loss of PLCη2 in PC12 cells attenuated the evoked fusion of vesicles recruited to the plasma membrane under enhanced Ca^2+^ influx conditions. PLCη2 was activated at cytoplasmic Ca^2+^ levels (∼800 nm) greater than those required to elicit maximal vesicle exocytosis (∼400 nm) in PC12 cells, which corresponds closely to the observed *in vitro* Ca^2+^-dependent activation of PLCη2 but not PLCδ1 (41). The hydrolysis of PI(4,5)P_2_ with DAG generation, F-actin disassembly, and the increased exocytosis of non-resident vesicles was also only evident at the higher Ca^2+^ concentrations. Extrapolation of these results into the nervous system suggests that loss of PLCη2 could slow neuropeptide secretion under conditions of high demand (*e.g.* sustained or high frequency stimulation), where higher Ca^2+^ levels are attained. A phenotype in the PLCη2 knock-out mouse (56) may only be evident under such conditions of stress. The current study indicates a functional role for PLCη2 as a Ca^2+^-dependent effector that regulates vesicle trafficking through its hydrolysis of PI(4,5)P_2_ and consequent remodeling of the actin cytoskeleton. The unanticipated localization of PLCη2 to F-actin supports such a role.

Compared with MS conditions, SS conditions uniquely activated PLCη2, PI(4,5)P_2_ hydrolysis, and F-actin disassembly and promoted a shift in the exocytic pathway toward new arriving vesicles without markedly altering the total number of vesicle exocytic events. PLCη2 activation both inhibited the exocytosis of resident vesicles and facilitated the exocytosis of vesicles trafficking to the plasma membrane during stimulation, as shown by PLCη2 knockdown. PLCη2 activation was also responsible for the F-actin disassembly promoted under SS conditions. However, unlike the effects of F-actin disassembly promoted by PLCη2 activation, halichondramide treatment mainly affected the fusion of non-resident vesicles without affecting the fusion of resident vesicles. The partial inhibition of resident vesicle fusion from PI(4,5)P_2_ hydrolysis might result from the inhibition of the local F-actin assembly, mediated by actin-associated proteins such as N-WASP and Arp2/3, that enhances vesicle exocytosis (57, 58). Because halichondramide causes F-actin disassembly without PI(4,5)P_2_ hydrolysis, it is possible that PI(4,5)P_2_-dependent F-actin assembly still occurs to support resident vesicle fusion.

The facilitation of the exocytosis of newly recruited vesicles by PI(4,5)P_2_ hydrolysis is readily understood as a consequence of the disassembly of a cortical F-actin meshwork that hinders vesicle access to the plasma membrane. This is consistent with the inhibition and stimulation of non-resident vesicle exocytosis by jasplakinolide and halichondramide, respectively. PI(4,5)P_2_ regulates numerous actin-binding proteins, such as scinderin (also known as adseverin), gelsolin, profilin, villin, and cofilin (24). Cofilin and scinderin are sequestered by PI(4,5)P_2_ and released upon PI(4,5)P_2_ hydrolysis to sever F-actin filaments (31, 45). Scinderin in chromaffin cells has been implicated in the Ca^2+^-induced disassembly of F-actin (27, 52). The Ca^2+^-dependent activation of PLCη2 and PI(4,5)P_2_ hydrolysis would release bound scinderin and related proteins to promote their actin-severing activity. Although PKC activation was also reported to promote cortical F-actin disassembly in chromaffin cells (59), we found that overexpression of a phosphoinositide 5-phosphatase that hydrolyzes PI(4,5)P_2_ without DAG generation closely mimicked the impact of PLCη2 overexpression on vesicle exocytosis (data not shown), which suggests that it is the loss of PI(4,5)P_2_, rather than an increase in DAG that is principally responsible for the cytoskeletal remodeling and changes in vesicle trafficking in PC12 cells. It was also recently reported that PI(4,5)P_2_-dependent F-actin remodeling was required for vesicle translocation to the plasma membrane in chromaffin cells (60).

PI(4,5)P_2_ is required for multiple steps in vesicle exocytosis, which suggests additional roles for PLCη2 as a Ca^2+^-dependent modulator. The priming factors CAPS and ubMunc13-2 are required for the regulated fusion of both resident and newly arrived vesicles (36). Studies in PC12 cells showed that the partial hydrolysis of PI(4,5)P_2_ by PLCη2 at elevated Ca^2+^ levels was sufficient to reduce the PI(4,5)P_2_-dependent activity of CAPS while maintaining the PI(4,5)P_2_-dependent membrane recruitment of Munc13 (9). Membrane-recruited Munc13 was further activated by DAG to compensate for the loss of CAPS activity (9). DAG production at elevated Ca^2+^ has been inferred to promote augmentation of synaptic neurotransmitter release mediated by Munc13-2 (20), but the PLC responsible for DAG generation had not been identified. Our results suggest that PLCη2 may mediate synaptic augmentation promoted by elevated Ca^2+^. The extensive role of PI(4,5)P_2_ in regulating plasma membrane-associated processes indicates that PLCη2 may have other functions in response to sustained Ca^2+^ elevations that are linked to the actin cytoskeleton, such as endocytosis, cytokinesis, and neurite outgrowth. The current work characterizes one of the roles of PLCη2 as a Ca^2+^-dependent regulator of the actin cytoskeleton and secretory pathway in neuroendocrine cells.

## Author Contributions

M. Y. and T. F. J. M. conceived the study and wrote the manuscript. M. Y. and D. M. K.-G. performed the experiments, analyzed the data, and prepared the figures.

## Footnotes

2 The abbreviations used are:

PI(4,5)P_2_: phosphatidylinositol 4,5-bisphosphate
PH: pleckstrin homology
PLC: phospholipase C
DAG: diacylglycerol
TIRF: total internal reflection fluorescence
BDNF: brain-derived neurotrophic factor
EGFP: enhanced green fluorescent protein
C1: protein kinase Cδ-C1 domain
MS: mild stimulation
SS: strong stimulation.
